# European Rural Development Policy Approaching Health Issues: An Exploration of Programming Schemes

**DOI:** 10.3390/ijerph16162973

**Published:** 2019-08-18

**Authors:** Antonella Samoggia, Aldo Bertazzoli, Arianna Ruggeri

**Affiliations:** Department of Agricultural and Food Sciences, University of Bologna, 40127 Bologna, Italy

**Keywords:** policy, health, nutrition, agriculture, food, environment, rural

## Abstract

Malnutrition, obesity, type 2 diabetes, micronutrient deficiencies, and the increase in non-communicable diseases are among the future European key challenges in health and welfare. Agriculture and rural development policies can positively contribute to a healthier and nutritious supply of food. The objective of the research is to analyze to what extent European 2007–2013 and 2014–2020 rural development programmes address the nexus between agriculture, food, health, and nutrition to respond to the evolving dietary needs. The research carries out a quali-quantitative content analysis on all 210 European rural development programmes. Results show that the interconnection between agriculture, food, health, and nutrition is present, with differences in the European agricultural and rural policy programming periods. The main interlinking issues of the nexus are food safety, food quality, diseases, nutritional aspect, animal health and welfare, plant health, and environmental health. Healthier and nutritious food-related issues are emerging, addressing dietary needs, and sustaining consumer food trends. Healthy and nutritious food is pursued by combating foodborne communicable diseases and non-communicable diseases. The future Common Agricultural Policy, including its rural dimensions, should support the consumption of healthy foods produced in ways that are environmentally and economically sustainable.

## 1. Introduction

The triple burden of malnutrition/undernutrition, obesity, and micronutrient deficiencies is an increasingly troublesome worldwide phenomenon. The upward trend of non-communicable diseases (NCDs) is one of the future key challenges in health and welfare. The World Health Organization (WHO) and other international organizations call for agricultural and food policies in order to ensure secure and sufficient supplies of safe and nutritious food [1,2,3,4]. The Food and Agriculture Organization of the United Nations (FAO) and WHO promote a worldwide approach, where agriculture is seen as the source of nourishment, thereby linked to health and dietary priorities [5]. The United Nations’ vision of the Sustainable Development goals for 2030 aims at “a world where food is sufficient, safe, affordable and nutritious”. More specifically, the United Nations’ Sustainable Development Goal 2 (SDG 2) focuses on Zero Hunger, seeking to “end hunger, achieve food security and improved nutrition, and promote sustainable agriculture” [6]. Food systems are the results of the combination of processes that link agricultural production to consumption, including the positive and negative impacts of the relevant activities on human and environmental health and wellbeing [7].

The European Union (EU) approved Article 168 of the 2007 Lisbon Treaty, stating that “A high level of human health protection shall be ensured in the definition and implementation of all Union policies and activities”. At the European level, there is awareness that the health condition of the population is the result of a number of policy areas, in particular social and regional policy, taxation, environment, education, and research. On this basis, the European Commission supports the Health in all policies (HIAP) approach, according to which health should be an integral element in most major EU strategic initiatives, such as its strategies for growth and jobs and sustainable development. While health and health equity have arguably attracted more attention within EC policy-making processes, particularly in light of concerns around climate change and the UN 2030 Sustainable Development Agenda, the emphasis has skewed towards internal markets, competition, and economic policies. Thus, more can be done to ensure that health implications are considered and taken into account in all policy areas.

The Common Agricultural Policy (CAP) is Europe’s funded policy priority. It accounts for around 408 Billion Euros in 2014–2020 programming period (2018, EU-28) [8]. If measured as share of the total EU budget, the CAP’s budget has decreased considerably over the past 25 years, from 73% (1985) to 39% (2015) [9], but still represents the EU key investment policy. The CAP impacts on European citizens’ health, providing healthy and nutritious food, and acting on animal welfare and environmental conditions. The CAP can ensure European citizens’ food supply in line with the evolving dietary needs [10,11,12].

The CAP includes the rural development policy that supports rural areas to meet economic, environmental and social challenges (so-called “second pillar”) and complements the system of direct payments to farmers and measures to manage agricultural markets (so-called “first pillar”). The current rural development policy’s main overarching priorities are fostering agricultural competitiveness; ensuring sustainable management of natural resources and climate action; achieving balanced territorial development of rural economies and communities including the creation and maintenance of employment [13]. At a member state level, the rural development policy is implemented through national and regional schemes of interventions called rural development programmes (RDPs).

European agricultural and rural policies have evolved since 2000. The CAP’s objectives are to manage the single EU market and to address a number of other objectives at the trans-national level, including food and feed safety, animal health and welfare, plant health, and public health as well as consumer interests. Agriculture and rural development policies can positively contribute to healthier life-preserving public goods, such as environment, air soil, land, water quality, and climate [14,15,16,17]. The 2011 CAP reform strengthened the economic and ecological competitiveness of the agricultural sector, promoted innovation, combatted climate change, and supported employment and growth in rural areas. More recently, the CAP has provided incentives for increasing fruit consumption and limiting provision of food with added sugars, salt, fat, and sweeteners or artificial flavors in schools. These incentives have also encouraged crop diversification as EU-level measures. These could help improve the quality of people’s diets. Among the recent interventions, the improved availability of fresh fruit and vegetable production, incentives for schools’ fruit schemes, and crop diversification, are recognized as important initiatives to improve nutrition for daily diets at European level [11,18].

Although health and nutrition issues are not officially included in the mainstream rural policy, it is interesting to explore their existence and definition in this policy domain. Past research explored the interconnections between agriculture, food, health, and nutrition (AFHN nexus). These aspects can be addressed within the multi-dimensional setting of rural development within the CAP. Thus, it is relevant to research to what extent European member countries’ national and regional RDPs refer to health and nutrition issues. The objective of the research is to explore if RDPs address the nexus between agriculture, food, health and nutrition. Governing bodies may converge or emphasize different issues relating to health and nutrition. An exploration of whether and how RDPs refer to issues related to health and nutrition can help to inform future debates on European policy reforms.

### 1.1. European Agricultural and Rural Policy Evolution

Since its foundation, the CAP contributed to assure the availability of food supplies and to respond to European dietary needs [17,19,20,21,22,23]. It was introduced in 1962 and contributed to the European population’s food security, by achieving an adequate quantity of nutritious food at affordable prices after the devastation of the Second World War. By the 1970’s, the CAP provided ‘fair living subsidies’ to farmers, increasing the levels of productivity which led to a surplus of food. From the 1980s through the early 1990s, the CAP imposed specific measures to manage the overproduction of food, with the objective of aligning agricultural production with market needs and supporting farmers. In the 1990s there was increased emphasis on food quality, protecting traditional and regional foods, and caring for the environment. In the 2000s, the CAP’s policy responsibilities were widened to include rural development, delivered through multi-annual programmes which focused on economic, social, and cultural development. Between 1990s and 2000s there was an abundant food supply and robust trade across Europe. During those years, there has been a wider spread of foodborne diseases and food safety scandals. The rich food offer contributed to over-nutrition, which in turn contributed to high rates of chronic and degenerative diseases, in particular NCDs, across Europe [10,24]. Currently, Europe is facing a dietary emergency connected to over-nutrition and overabundance of unhealthy food, with adverse effects to population health and to society in general, as well as the financial sustainability of the healthcare systems. This has imposed high health service, economic, and societal costs. This double burden of malnutrition is forcing European member countries to focus on a preventive approach and ex-ante investments in order to contain food-related health problems and improve health and nutrition practices of European citizens. Societal needs are fast evolving and the European countries need to place an additional priority on daily dietary needs.

During the period 2007–2013, rural development gained a specific and more strategic role within the programming strategy [25]. RDPs are developed according to key objectives focused on competitiveness, environment and land management, and improved quality of life. The ongoing strategy, for the period 2014–2020, identifies producers’ and consumers’ needs within a stronger and more holistic commitment towards a sustainable approach characterized by key objectives: “viable food production, sustainable management of natural resources and climate action and balanced territorial development” [10]. RDPs absorb around 100 Billion euro for the 2014–2020 period, corresponding to 24.4% of CAP budget (2018, EU-28) [8].

The European Commission’s acknowledgement of the relation between CAP, rural policy and the health of European citizens is increasing. The European Commission in “The Future of Food and Farming” Communication [26] states that “the CAP is one of the EU policies responding to societal expectations regarding food, in particular concerning food safety, food quality, environmental and animal welfare standards” and “the CAP also has a role to play in promoting healthier nutrition, helping to reduce the problem of obesity and malnutrition, making nutritious valuable products”. Among the policy agenda orientations, there is a call for strengthening rural value chains as means to respond to consumers’ demand for healthy food [26,27]. European Union policies can promote integrated approaches to developing rural value chains and local productive networks. These can effectively respond to “consumers’ demand towards healthy and quality of food products, and agricultural production processes” [26].

Furthermore, the European Union is aware of the key role that the CAP and rural policy can have in the global economy. The EU is the world largest agri-food exporter. Understanding global value chains’ market expectations can help the EU agri-food sector increase the exports. This can be achieved by stimulating more sustainable production and processing practices, better matching supply and demand, responding to the dietary changes, reducing food waste and food losses, and promoting a circular bio-economy [26]. The European Commission states that “the most important role for the policy is, therefore, to help farmers anticipate developments in dietary habits and adjust their production according to market signals and consumers’ demands” [26].

### 1.2. The Link between Agriculture, Food, Nutrition, and Health in the Literature

The link between agriculture, food, nutrition and health is increasingly explored by the academia, international organizations, and the private sector [1,2,3,16,17,28,29,30]. Recent academic papers review methods and tools used for assessing the implementation of government policies to create healthy food environments [31,32]. Healthy food environments aim to prevent obesity and diet-related NCDs, such as type 2 diabetes, heart disease, cancer, by providing food that contributes to preventing diseases, reducing risk, and improving human health.

There is increasing research that focuses on how EU’s policy approaches contribute to improving environmental health and reducing health inequality, within a wider public health perspective. Many publications in the field of public health and nutrition discuss the role that agriculture plays in improving nutrition [3,12,16,17,18,24,28,29]. Other research studies have proposed the development of a framework to monitor government policies and actions for creating healthy food environments [33], so as to understand the impacts of agriculture and food system policies on nutrition and health [34]. Other studies point out the lack of political priority given to nutrition-related issues within the CAP [11] and the difficulties in the policy integration between nutrition and sustainability [35].

In addition, numerous studies point to other ways in which agricultural land can benefit health, such as providing access to greenspace and natural outdoor environment. Accessibility and time spent in outdoor environments, combined with healthier food habits, have been found to reduce the risk of obesity [36,37,38,39]. Academics and policymakers have promoted a nutrition-sensitive approach in agricultural and rural policy in developed and developing countries, mainly aimed at reducing malnutrition and increasing access to healthy nutritious food [40,41,42,43].

The attention to NCDs suggests a new approach towards food and agriculture at European level [16,17,44,45,46]. Past studies show that linking agricultural and rural policies to health-related issues contributes to increasing food availability and affordability and to promoting healthier diets, which reduce NCDs [44]. The literature recommends agricultural policies, aiming to develop actions that involve various agri-food system actors (e.g., farmers, food processors, retailers, consumers, and other economic agents), and target different key beneficiary groups, such as women, children, sick or elderly people [28,34,40,47]. The policy actions may aim to modify the behavior of agri-food system actors mainly through incentives and regulations, and to promote interventions to address health and nutritional damages occurred due to inappropriate food behaviors [48]. The food and beverage processing and retail industries and the international supply chains are the key actors in shaping people’s diets, as well as farmers’ production decisions and consequent incomes. There is growing consensus that the food system should be addressed considering the whole chain from farm to plate within a health-sensitive and sustainable European food and agricultural policy [49].

Agricultural economics and policy experts at the European and international levels start conceptualizing the need to develop a broader agricultural and food policy to provide healthy and safe diets for Europe and the world [50,51]. There is a call to start “thinking out of the box” [52] when envisioning the CAP to be reformed and implemented after 2020. Experts support that the European food system challenges are to be approached addressing the entire food system, including the agricultural sector and the relevant policies. The challenges of over-consumption and consequent health diseases put pressures on the farm system [52]. Prominent scholars and experts in addressing European environmental and agricultural challenges support that the CAP has to adapt to favor sustainable food production and consumption systems. The solutions should respond to the growing incidence of obesity, diabetes and other non-communicable, lifestyle-related, ill health [52]. A similar process is explored for the fishery policy, highlighting opportunities for enhancing healthy diets within a multi-sectoral policy [53,54]. So far there has been limited debate in the literature on how health policy and agricultural policy can be implemented consistently, how to develop a multi-sectoral approach that considers various underlying causes of malnutrition, and how to ensure that agricultural policy focuses concretely on the nutritional quality of what is being produced, in order to create a positive impact on human health [16,17,55]. A robust debate on the role of rural development policy in supporting healthy and nutritious food accessibility could contribute to making the health and nutrition challenge in a progressive manner. It would contribute to a constant identification of societal priorities and establishment of a common policy-making process.

Past research support that the focus on the health impact of the policies varies. The increasing consumers’ interest in a healthier and more environmentally sustainable way of life is more clearly supported by the health and environmental policy areas, in contrast to rural and agricultural policy areas. The latter are not sufficiently engaged in helping to achieve these outcomes. There remains a disconnect between a key societal trend focused on healthier and environmentally-oriented lifestyle and the most relevant policy areas. Moreover, previous studies support a need for stronger cooperation among the different agri-food system actors to improve food security and nutrition in our societies [11,56,57,58,59,60,61]. Therefore, it is necessary to research on effective synergies among health, nutrition and sustainable agriculture [16,17,30,62]. Past research supports that health and nutrition are not priorities for agricultural and rural policy development. More particularly, health and nutrition issues are not adequately addressed in the priorities setting of the ex-ante phase, monitored and assessed during the agricultural and rural policy implementation, and evaluated in the ex-post phase of the policy cycle [63,64,65,66,67].

Latest participatory research processes involving farmers, food entrepreneurs, civil society activists, scientists, research scholars, and policy-makers at European level are proposing a Common Food Policy for the EU [68]. The aim is “to address climate change, halt biodiversity loss, curb obesity, and make farming viable for the next generation”. These objectives can be achieved by aligning various sectoral policies affecting food production, processing, distribution, and consumption, asking for a clear transition to sustainability. Thus, they propose a new governance architecture for food systems that finds a correspondent new European institution governance setting, with inter-directorate cooperation coordinated by a dedicated European Commission official [69].

Recent studies support that an EU Food Policy would help address the issue of European food surpluses and low prices for farmers [50]. There is a call for a reframe of agricultural policies to shift emphasis from high volumes of outputs to high diversity of crops and nutritional quality of foods produced. The aim is to reorient agricultural priorities from producing large quantities of food to producing healthy food. This founds on the belief that “agriculture is a core determinant of nutrition” [30]. Agricultural policies can contribute to enhance nutrition outcomes.

Finally, recent review studies support that agricultural, trade and consumer policies have the potential to impact diets and nutrition, even if they are not explicitly designed for such purpose. There are two main approaches to influence diets: increasing the income, and changing food availability and/or relative food prices and/or preferences for food. However, there is need to improve the evidence base. This may focus on quantitative evidence based on rigorous study designs, carried out in cooperation between public health and agriculture economy scientists [70]. Standardized or harmonized indicators would ensure consistency and robust applicability purposes to support, implement, and monitor relevant policies [7].

## 2. Materials and Methods

**Figure 1:**
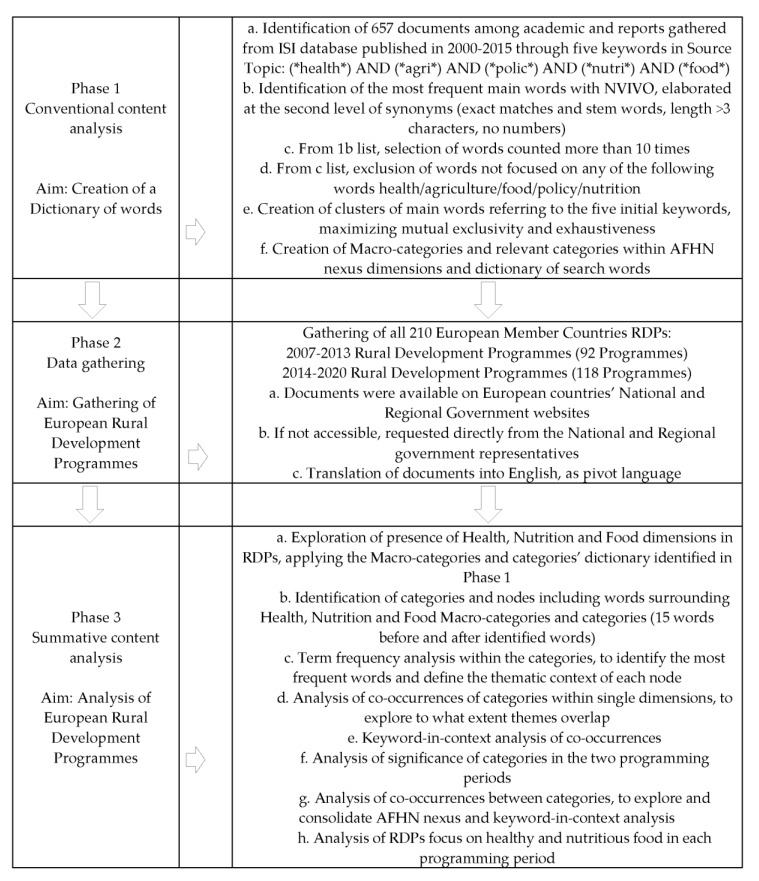
Research Phases.

The methodological approach aimed to explore the presence of the nexus between agriculture, food, health and nutrition in the rural development programme documents. The research applies a content analysis methodology in three phases (Figure 1). Phase 1 set the dictionary of words of AFHN nexus, as identified in the relevant literature. Phase 2 gathered and prepared the RDPs. Phase 3 investigated the existence and frequency of the AFHN nexus in European Member states RDPs. The content analysis identified whether the documents contain the word categories, what issues they are associated with, and whether there are co-occurrences among word categories.

Phase 1 consolidated the AFHN nexus and detailed the concept identifying the relevant words, based on the academic paper and literature review present in ISI Web of Knowledge database (Figure 1). The concepts of AFHN nexus was searched by including the following wild cards in the Topic section: *health*, *agri*, *polic*, *nutri* and *food*, identifying 657 sources. The abstracts of these sources were elaborated with the Conventional content analysis methodology [71] with the support of NVIVO, a qualitative data analysis software (QSR International, 10th version), to identify the most frequent terms. The software elaborated the words at the second level of synonyms. From this list only the words that appeared more than 10 times were kept. Then terms referring to single countries or specific regions, nonspecific verbs, adverbs, numbers, or other generic words were excluded. The consolidated list included health/agriculture/policy/nutrition focused words. The study’s researchers agreed on how to cluster the terms with the aim of creating categories tightened up to maximize mutual exclusivity and exhaustiveness [72]. Categories were associated with one of the AFHN nexus dimensions and grouped into macro-categories if needed. The output of this phase was the creation of the dictionary for each nexus dimension, to be used in the second phase (Table A1, Table A2 amd Table A3).

Phase 2 aimed at gathering all 210 European RDPs for the periods of 2007–2013 and 2014–2020 (Figure 1) (Table A4). The programme documents came from the European Commission, national, and regional government websites. They are mostly written in each country’s respective language. Thus, when necessary, the source texts were translated with Google Translate (GT) into English, adopted as pivot language. This is a well-established practice accepted by the European Commission [73]. GT provides translations that between “European languages are usually good” and the “existing language translation algorithm is constantly improved” [74,75,76,77,78,79,80,81]. The consistency, clarity, and appropriateness of the languages of a homogeneous corpus of official technical documents, such as the RDPs, would further improve and standardize translated outputs.

Phase 3 was based on a Summative content analysis [82,83] (Figure 1) aimed at identifying the presence and the frequency of the macro-categories and categories identified in Phase 1, referring to Health, Nutrition, and Food dimensions in the RDPs. The keywords became categories’ nodes, customized to 15 words before and after searched term. Nodes were checked confirming and contextualizing the meaning of the words identified to assess the consistency with the corresponding category. Then the research carried out a term frequency analysis of the nodes, with stem words at first level of synonym, to identify the most frequent terms, explore the thematic context of each node, and carry out a first relevance of the AFHN nexus. The term frequency analysis counts the times of each word and synonyms. Then it calculates the corresponding weighted percentage, that is the frequency of the word relative to the total words counted. The weighted percentage assigns a portion of the word’s frequency to each group. This step provides a first focus on the quali-quantitative content analysis of the documents’ nodes. Then an analysis of co-occurrences of categories within single dimensions consolidated categories within Health, Nutrition and Food dimensions, to explore the extent to which themes overlap and create conceptual concentration. The analysis of co-occurrences was completed with a keyword-in-context analysis to confirm dimension, macro-category, and category consistency. The software elaboration was carried out keeping track of the documents’ country. This allowed a synthetic view of the findings at a country level. Then, there was a focus on the single categories in the two programming periods, to explore whether there was a change in the thematic priorities over time. To explore and consolidate AFHN nexus, the research explored the co-occurrences between categories of all three dimensions, supported with a keyword-in-context analysis to confirm and contextualize meaning. The elaboration was carried out with NVIVO software. Finally, there was an analysis of the focus on healthy and nutritious food issues in each programming period in each of the 210 RDPs carried out with SPSS software. The tables exclude non-identified categories.

## 3. Results

The interconnection between agriculture, food, health, and nutrition is present in the rural policy programming schemes, with differences in the two programming periods. There is higher prominence on health, with focus on animal health and welfare, plant health, and environmental health, compared to health, related to nutritional issues, such as food safety, food quality, diseases, nutritional aspect. Healthier and nutritious food-related issues are emerging, addressing dietary needs and sustaining consumer food trends. Member countries’ RDPs show that the AFHN nexus varies from one country to the next, but it is relatively constant over time within the same country.

### 3.1. Programming Issues within Single Nexus Dimension

#### 3.1.1. Health

The RDPs address the Health dimension focusing on Health, Safety, Diseases, and Medical categories (Table A5). The Health macro-category is mostly focused on a healthy environment for animals and people. In detail, the Health category (9037 times) mainly focuses on the agricultural sector and is often associated with animal, environment, plants, agriculture, protection, development, and farms. It is also connected to welfare, quality, and food. The Unhealthy category, even though rarely mentioned (12 times) is more strongly associated with food quality, energy content, and naturalness. Within the Safety macro-category, it is clear that rural policy addresses the issue of risk, prevention, and safety. Countries aim for a rural development policy to set up initiatives to manage, measure, implement, control risk, and to prevent damage, risk, disasters, fires especially of forests and on nature. The Safety category is more clearly directed to improve food quality and safety. The RDPs focus on human health and food when they refer to hygiene emphasizes animal, welfare, safety standards and environment. The Diseases macro-category mostly focuses on preventive interventions for the benefit of plants, animals, forests, to prevent pest and other natural calamities. There is also a focus on disabled people and persons rights and equality. There is limited attention to non-communicable and single diseases. Few programming schemes focus on type 2 diabetes and cardiovascular diseases. When mentioned, the RDPs aim at tackling them because considered health challenges. Finally, within the Medical macro-category, the documents target pharmaceuticals focusing on medicinal aromatic plants, crops, herbs, fruit, vegetables, animal, and food. The RDPs are also directed towards medical services, healthcare, community infrastructures, education, and schools. In synthesis, the Health dimension focuses on agriculture and environment, in particular plant and animal health; however, there is emerging interest towards healthy food, food safety, and hygiene.

The Health categories and concepts are variously overlapping. In the documents there is a conceptual connection between the categories of health and safety, risk and diseases (Table A8). The Diseases category is often associated with infection and epidemiological phenomena, specifically referring to bacteria and pathogens. The safety concept is also associated with hygiene and prevention of risk.

**Figure 2:**
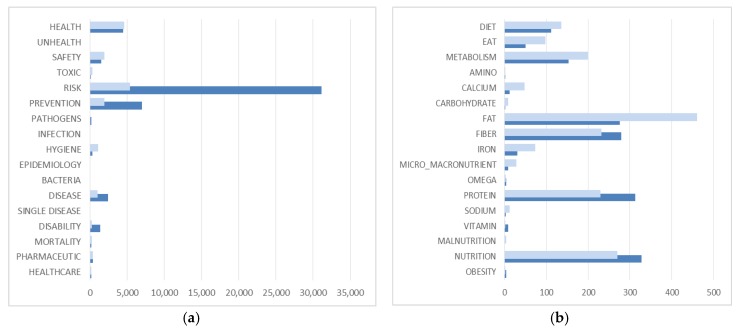

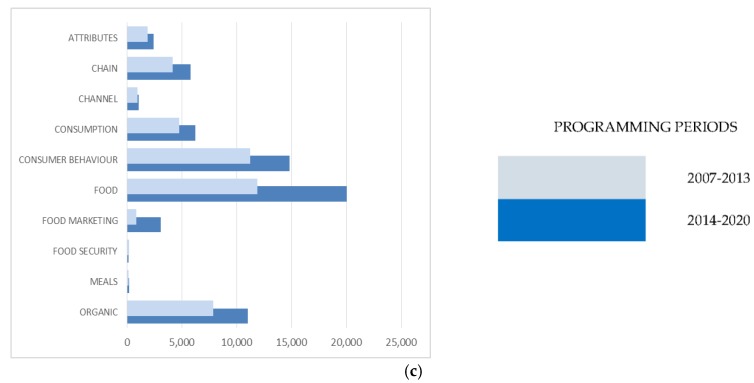
Frequency of each category in the programming periods (**a**) Health categories (**b**) Nutrition categories (**c**) Food categories.

Finally, the documents refer to mortality issues together with single diseases, such as malaria, obesity, diabetes, and cancer. The results show that there are differences in the two programming periods (Figure 2). The 2007–2013 period had a rural policy that stressed hygiene more than the following programming period. The second period focuses on risk, diseases and disability. Italy, Finland, Czech Republic, Spain and Wales are the most sensitive countries to those issues.

#### 3.1.2. Nutrition

The RDPs address the Nutrition dimension focusing on nutrition in general, nutrients, and dietary aspects (Table A6) The Nutrition macro-category refers to the nutritional quality of animal feed products and their impact on health. Similarly, the malnutrition category mainly focuses on animals. The Nutrition macro-category refers to obesity category, that is associated with human health. The aim is to reduce the incidence and rising levels of obesity, across all age groups. Furthermore, animal health is also the main element of the Dietary macro-category. The RDPs focus on animal feed and digestion, as well as anaerobic digestion, organic products, and water use in agriculture. The Diet category focuses on diet, healthy and quality food, and mentions cow feed quality. The Nutrients macro-category includes micro and macronutrients, nitrogen, fertilizers, phosphorus, associated with soil and water. The Proteins category is referred to in association with crops, cereals, animal feeding, oilseeds, plants, and legumes. The Fat category refers to animals and animal products, especially pigs, cattle, oils, and dairy. With respect to fibre the documents refer to infrastructures. RDPs do refer, although infrequently, to vitamins and omega (3 or 6), associating them to food content, substances, nutritional quality, and health properties

There are limited co-occurrences among the nutrition categories (Table A9). The most significant are nutrition and vitamin, omega and vitamin, protein and amino acid, nutrients, and calcium. This shows an attentive and elaborated approach towards nutritional issues. RDPs focus on changes in nutritional aspects over time (Figure 2). The 2007–2013 programming period focuses on metabolism, eating and fat component of food and feed, whereas the following programming period more strongly addresses issues of protein and nutrition categories. The countries that mostly focus on nutrition dimension are Finland, Germany, Hungary, Sweden, and Scotland.

#### 3.1.3. Food

RDPs address the Food dimension focusing on food attributes, consumer, food chain system, and food security (Table A7). The Food macro-category is positioned within an agricultural framework, in connection with food and agricultural sector development, food processing industry, promotions, markets, chains, and forestry. There is attention to food attributes, such as labelling, traceability, quality, system organization and regulations. Organic food products are mentioned in connection with food quality, market development, and farms. The programmes also refer to rural tourism, including meals, bed and breakfast, travelling, and accommodation. The Consumption macro-category focuses on environmental and food consumption. The programmes address the issue of energy and water consumption and their environmental impact. They also pay attention to food consumption and consumer behavior, focusing on an increase in food quality and markets. There is interest also in consumer purchasing, awareness, and quality increase. Similarly, they refer to promoting food products, taking into account various food system dimensions, including food market, food chain, and organization. The Food chain macro-category highlights a key aspect of rural development programmes. Food chains are connected to food supply and markets, as well as promotion of local development, value chain, and short food chain. There is also attention on food sales, retailing, and trading. Furthermore, the programming documents address the Food security macro-category, associated with improving food quality, health and safety, of food and agricultural sectors. There are significant co-occurrences among food categories. In particular, the food category co-occurs with chain, consumer, channels, and attributes categories (Table A10). Finally, the 2014–2020 programming period is more focused on most numbers of categories of food, compared to the previous programming period (Figure 2). The countries that most significantly refer to food in rural policy are Italy, Spain, Wales, and Slovenia.

### 3.2. Countries’ Focus and Priority Evolution Over Time

The above analysis shows that the words health and nutrition are used variously to address the issue of healthy and nutritious food. There are categories that clearly target the core issue, whereas others are only partially linked to healthy and nutritious food. Thus, at this stage, the analysis focuses on what emerged as strongly consistent with the core AFHN nexus concepts, to identify if and to what extent the single regional governments referred to healthy and nutritious food in RDPs in the 2007–2013 and the 2014–2020 programming periods.

**Figure 3:**
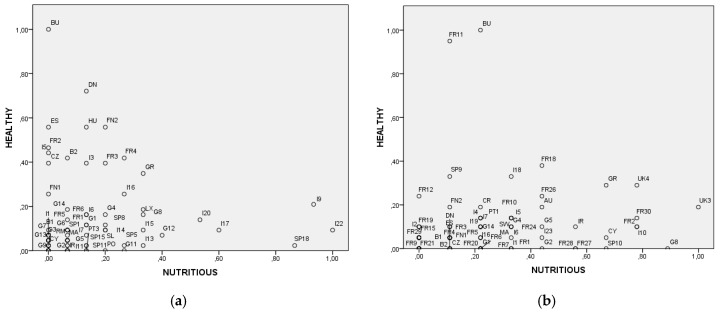
Healthy and Nutritious focus of Rural Development Programmes. (**a**) 2007–2013 Rural Development Programmes (RDP): Bulgaria (BU) RDP had the highest value of Healthy Food focus with 43, and a 2007–2013 Italian (I22) RDP had the highest value of Nutritious Food focus with 15 (**b**) 2014–2020 Rural Development Programmes (RDP): Bulgaria (BU) RDP had the highest value of Healthy Food focus with 21, and a 2014–2020 British (UK3) RDP had the highest value of Nutritious Food focus with 9.

For each RDP and each programming period, the Healthy and Nutritious values were calculated as Figure 3. For Healthy, it was the sum of the categories and categories’ co-occurrences of food and food-related words or nutrition and nutrition-related words. For Nutritious, it was the sum of the categories and categories’ co-occurrences of food and food-related words or health and health-related words mentioned. The values obtained were then normalized maximizing to 1 the highest RDP value.

The results show that Bulgaria is the country with the highest focus on Healthy food issues, in both programming periods. On the other hand, an Italian RDP and a British RDP had the highest attention on Nutritious food respectively in the 2007–2013 and the 2014–2020 programming periods (Figure 3). Overall in the first period regions mostly addressed Healthy food issues, whereas, in the second, regions primarily focused on Nutritious aspects. In the 2007–2013 period, the regions that most incorporated the AFHN nexus were Denmark, Estonia, Hungary, Finland, and France, which focused on Healthy issues, and Italy and Spain which focused on Nutritious. In the 2014–2020 period, the countries more strongly focused on Healthy elements are France, Spain, and Italy. The United Kingdom, Germany, France, Italy, Greece, Cyprus, and Spain focused on Nutritious. It is interesting that in no programming period did any country strongly focus on both aspects.

## 4. Discussion

European rural development programming addresses policy priorities consistent with the AFHN nexus. Healthy and Nutritious Food is a cross-cutting programming issue with different levels of political recognition, implemented through a number of interrelated policy measures. The research results show that rural policy interprets agricultural production as the synthesis of human, animal, and environmental health, with a key role in providing healthy and nutritious food for Europeans. The RDPs analysis shows that rural policy is simultaneously aimed at ensuring a number of preconditions for environmental and animal health, and at delivering healthy and nutritious food. The research results suggest that there are two primary approaches towards healthy and nutritious food in rural development policy. The first approach is aimed to ensure healthy and nutritious food by preventing foodborne communicable diseases. The other approach is to provide healthy and nutritional food by combating non-communicable diseases. This latter approach is emerging but remains less critical.

First, preventing foodborne communicable diseases is a priority in RDPs. In that regard, food safety is the forefront policy, with a clear and long-established programming legitimacy. The research findings show that its implementation includes food, animal, and plants health, in particular delivering safe and nutritious food, information on origin, labelling, healthy animal feed and high standards of animal health and plant protection. Food safety aims to prevent the use of chemicals hazardous to humans, animals, and the environment. Due to the consequences of food scandals from 1990s, member countries have invested in food safety in the RDPs of both programming periods. In 2002 the EU has established a dedicated Agency, the European Food Safety Authority, with the responsibility for risk assessment. Food Safety issues have become more prominent following the membership of new countries from East Europe, characterized by less developed rurality systems compared to Western European countries. Eastern European countries have mostly focused their RDPs on this topic.

Second, the findings indicate that the approach towards combating non-communicable diseases is of more limited importance to regional governments, compared to foodborne communicable diseases prevention. RDPs start including actions to address the increase in NCDs and to combat unhealthy lifestyles and unhealthy nutrition habits. The nutritional perspective adopted takes into account obesity, nutrition, diet, as well as malnutrition, diseases, and unhealthy food habits. RDPs state that the main concerns relate to inadequate attention to healthy eating, insufficient physical activity, and unhealthy lifestyle and diet. RDPs highlight the issues of food consumption, and of the kinds of and the quality of the food production, in relation to human health and nutrition. Given that food security is fairly assured, food needs are changing, and it is necessary to support changing dietary preferences and lifestyles in Europe.

RDPs may be used to intervene on public health, and to raise awareness that food habits play an essential role in life expectancy, premature mortality, and quality of life. The provision of healthy food and higher food quality are necessary “for the sake of population health”. There is awareness that unhealthy life and eating habits can lead to overweight and obesity. Thus, RDPs support the promotion of healthy, high quality, highly nutritional food products, and environmentally sustainable food production and commercialization consistent with preserving the environment and animal welfare. The objective is to ensure rural areas’ sustainability and to preserve the environment, farmers’ working conditions, traditional landscape, and biodiversity. The key risk factors, such as unbalanced diet and physical inactivity, may be tackled with the support of rural policy. The rural policy programming relies on the awareness that the establishment and maintenance of a healthy lifestyle depend upon individuals’ choices, as well as on the environment within which the choices are made.

Finally, research results support that the RDPs’ consideration of healthy and nutritious food provision takes into account current dietary trends and consumer health-oriented food behavior. There is an increasing awareness and demand amongst consumers of what is nutritious and healthy, and therefore growing demand for such foods. This leads to heightened awareness of food selection, consumption and purchase. Thus, it may be appropriate to involve the food production and the food distribution actors in the rural development programming, in order to take advantage of retailers’ critical role in consumers’ accessibility to healthy food.

## 5. Conclusions

The CAP’s capability to support directly health and nutrition-related issues can be strengthened. Currently, it lacks a clear healthy nutrition approach, due to an undefined inclusion of these issues in the CAP’s regulations, and possibly because of limited capability to invest monetary resources. Moreover, the European citizens’ food security at short to medium-term can be considered consolidated. At its inception, health and nutrition were the CAPS’s central issues, given its purpose of ensuring European citizens’ food security. Although this has now been consolidated, it should not be taken for granted. The EU should not underestimate or neglect the risk that financial and economic crises (e.g., volatility of food and food inputs’ prices) or environmental phenomena (e.g., climate change, water scarcity, and pests and diseases) may have on the European food supply.

Over time, the concept of rurality has been widening to include other policy dimensions. Its capability to evolve and adapt to upcoming challenges means it continues to be acknowledged as a key policy area at European level. The CAP has successfully ensured food in quantity for Europe in response to food scarcity, promoted environmental respect in response to ecological crises and climate change, and made sure that farmers had adequate economic compensation to achieve a good living standard.

Currently, Europe is facing a new challenge, with increasing healthcare costs due to unhealthy lifestyles. Agriculture and rural policies can effectively contribute to a preventive approach to food-related diseases in response to what is a public health epidemic. Health and nutrition should be included and championed within the wider set of policy priorities in the CAP’s future. Healthier food must be easily accessible. Policymakers should guarantee healthy, safe, environmentally-sustainable, and tasty food, taking into account all food system actors including agri-food producers, processors, retailers, and consumers.

More may be done to mainstream health considerations into other areas, like agriculture and rural development policy. The original mandate of the CAP was to achieve food security through agricultural interventions, hence the policy’s title. If the overall mission is to promote environmental protection, sustainable development, viable livelihoods for farmers, and healthy food, there is need to realign the principles and policies of the CAP and, perhaps, its nomenclature.

The worldwide experience of attempts to address health and nutrition highlights the challenges in developing and implementing well-grounded and evidence-based policies including nutrition-sensitive agriculture. [67]. At the European level, there remains an absence of an agricultural and rural development policy approachable to address health and nutrition-related aspects at national or regional level [42,45,48,62].

To conclude, despite little has been done in CAP to focus on the nutritional value of the food being produced to obtain a comprehensive public health perspective, academics and policymakers should address these issues and the societal challenges linked to obesity and other diet-related non-communicable diseases. The CAP, particularly its rural dimension, can be at the forefront of policies contributing to improved health. The CAP as a whole, including RDPs, should aim for EU food production and consumption that are healthy, nutritious, environmentally and economically sustainable, whilst remaining affordable and diversified.

This research adds to the understanding of health and nutrition in relation to rural programming policy. Protecting public and environmental health and providing sufficient nutritious and healthy food are increasingly being acknowledged. This is timely given the ending of the current programming period and the opening of discussions on the future of the CAP and consequent reform.

This research is based on secondary sets of information including programming documents. Direct interviews with policymakers who contribute to the definition of policy priorities at a regional level would provide further information on the policy process and the challenges in reconciling different, and at times conflicting, stakeholders’ expectations. Accordingly, future research should expand the programming documents analyzed, including other European funding, such as the European Social Fund, the European Regional Development Fund, and the European maritime and fisheries fund. The system actors and the policymakers working on agri-food come from different economic sectors and political backgrounds and should be aware of, and adopt, a multi and inter-sector approach. The success of this is based on flexibility and open-mindedness, together with a vision for, and knowledge of, the environmental, social, economic, and health context.

## Appendix A

**Table A1:** Dictionary of Health dimension: Macrocategories, categories, words.

Macro-Categories	Categories	Words
DISEASE		
	DISABILITY	disable*, disabil*
	DISEASE	diseas*, illness*
	SINGLE DISEASES	Malaria*, HIV, diabetes, cardiovascular, cancer*, NCDs, chronic, deficiency
	MORTALITY	mortal*, death*, deadly
HEALTH		
	HEALTH	healthy, healthier, healthful, health
	UNHEALTH	Unhealthy*
MEDICAL		
	HEALTHCARE	medical*, healthcare*
	PHARMACEUTIC	pharmaceutic*, medicin*, drug*
SAFETY		
	BACTERIA	Bacteri*
	EPIDEMIOLOGY	epidemiological*, epidemic*
	HYGIENE	hygiene*
	INFECTION	Infectious, infection, infecti*
	PATHOGENS	Pathogen*
	PREVENTION	prevention*
	RISK	risk*
	SAFETY	safet*
	TOXIC	tox*

**Table A2:** Dictionary of Nutrition dimension: Macrocategories, categories, words.

Macro-Categories	Categories	Words
DIETARY		
	DIET	Diets, dietetic, dietary, diet
	EAT	eat*
	INDIVIDUAL_METABOLIC	Metabolic, intake, digestion
NUTRIENTS		
	FAT	fat*, lipid*
	FIBER	fiber*
	IRON	iron*
	MICRO_MACRONUTRIENT	micronutrient*, macronutrient*
	NUTRIENT	nutrient*
	PROTEIN	protein*
	AMINO	amino*
	CALCIUM	calcium*
	CARBOHYDRATE	carbohydrat*
	OMEGA	omega*
	SODIUM	sodium*
	VITAMIN	vitamin*
NUTRITION		
	MALNUTRITION	malnutrit*, malnourish*
	NUTRITION	Nutritious, nutritionists, nutritional nutrit*
	OBESITY	obes*, overweight*, overnutrit*

**Table A3:** Dictionary of Food dimension: Macrocategories, categories, words.

Macro-Categories	Categories	Words
FOOD		
	FOOD	food*
	ATTRIBUTES	transgenic*, traceability, seasonal, novel, label*, convenience, affordability
	MEALS	snack*, meal*, breakfast*, lunch*
	ORGANIC	organic* (see Notes)
FOOD CONSUMER		
	CONSUMER BEHAVIOUR	purchas*, perception*, motivation*, lifestyle*, cognitive*, behavior*, awareness*, attitude* (see Notes)
	CONSUMPTION	consum*
	MARKETING	promotion*, advertising, marketing
FOOD SECURITY		
	FOOD SECURITY	“food security” (see Notes)
FOOD CHAIN		
	MARKET CHANNEL	vending*, store*, retail*
	FOOD CHAIN	chain*, intersectoral*

Notes: (i) Consumer behavior category created by searching the listed words within a category Food Consumption created with word Consum*. (ii) Organic category created by searching the listed word within the Food Category. (iii) Food Security category created with string Food Security.

## Appendix B

**Table A4:** Rural development programmes analysed.

Country	2007–2013			2014–2020		
	National	Regional	Total	National	Regional	Total
AUSTRIA	1		1	1		1
BELGIUM		2	2		2	2
BULGARIA	1		1	1		1
CEZCTH REPUBLIC	1		1	1		1
CROATIA			0	1		1
CYPRUS	1		1	1		1
DENMARK	1		1	1		1
ESTONIA	1		1	1		1
FINLAND		2	2		2	2
FRANCE		6	6	3	27	30
GERMANY	1	14	15	2	13	15
GREECE	1		1	1		1
HUNGARY	1		1	1		1
IRELAND	1		1	1		1
ITALY	1	21	22	2	20	22
LATVIA	1		1	1		1
LITHUANIA	1		1	1		1
LUXEMBOURG	1		1	1		1
MALTA	1		1	1		1
POLAND	1		1	1		1
PORTUGAL	1	3	4		4	4
ROMANIA	1		1	1		1
SLOVAKIA	1		1	1		1
SLOVENIA	1		1	1		1
SPAIN	1	17	18	2	17	19
SWEEDEN	1		1	1		1
THE NETHERLANDS	1		1	1		1
UNITED KINGDOM		4	4		4	4
Total	23	69	92	29	89	118

## Appendix C

**Table A5:** Word frequency within single Health dimension categories.

**Macroc.**	**Category**	**Freq.** **Categ.**	**1**	**2**	**3**	**4**	**5**	**6**	**7**	**8**	**9**	**10**
**HEALTH**	Health	9037	health	animal	environ.	products	plants	agricult.	welfare	areas	services	improv.
		WP	5.10	1.43	1.21	1.07	0.83	0.68	0.64	0.63	0.62	0.58
	Unhealthy	12	unhealthy	energy	areas	health	product	quality	food	industr.	natural	potentially
		WP	4.92	2.05	1.64	1.64	1.64	1.64	1.23	1.23	1.23	1.23
**SAFETY**	Safety	3489	safety	product	food	improving	quality	working	animals	environ.lly	health	using
		WP	5.37	2.14	2.10	1.30	1.15	1.04	1.02	0.98	0.96	0.84
	Toxic	479	toxicity	product	using	plant	protect.	harmful	classif.	pesticides	toxicol.	substances
		WP	5.80	2.91	2.67	1.48	1.31	1.28	0.92	0.74	0.68	0.63
	Risk	36483	risk	measuring	implements	controls	manag.	types	operators	areas	agricult.	relativ.
		WP	6.21	2.81	1.36	1.11	1.07	0.97	0.93	0.87	0.78	0.69
	Prevention	3172	prevents	forests	fires	disaster	nature	manag.	damage	risk	measuring	areas
		WP	5.32	2.79	2.08	1.68	1.61	1.60	1.46	1.34	1.24	0.98
	Pathogens	287	pathogens	plants	forest	prevent.	pests	climate	protect.	product	damage	increase
		WP	5.37	1.40	1.35	0.97	0.90	0.70	0.68	0.68	0.65	0.61
	Infection	132	infection	disease	animal	infectious	use	control	prevent	products	spread	causes
		WP	4.27	2.37	2.15	1.35	1.06	1.02	0.80	0.69	0.66	0.62
	Hygiene	1423	hygiene	animal	improving	welfare	products	safety	standards	environ.	conditions	environ.al
		WP	5.09	3.00	2.47	2.20	1.76	1.24	1.20	1.17	1.12	1.10
	Epidemiology	73	epidemics	diseases	production	risk	epidem.	health	plants	control	systems	organism
		WP	3.69	1.63	1.63	1.56	1.42	1.14	1.07	0.92	0.85	0.78
	Bacteria	133	bacteria	waters	plants	diseases	fungi	bacteriological	pollut.	organisms	products	cause
		WP	3.28	1.09	0.95	0.85	0.71	0.67	0.67	0.63	0.63	0.56
**DISEASES**	Disease	3509	diseases	pests	plants	organizations	scient.	prevent.	disasters	animals	forest	case
		WP	5.52	2.25	1.71	1.49	1.40	1.36	1.36	1.13	1.09	0.92
	Single Diseases	71	cancer	diseases	agent	communicable	prevention	pino	oaks	products	agricult.	farms
		WP	2.86	2.28	2.08	1.76	1.17	1.04	0.78	0.78	0.72	0.65
	Disability	1631	disabled	persons	rights	people	nations	implementing	convent.	applicat.	funds	policy
		WP	6.50	2.46	1.79	1.40	1.40	1.30	1.28	0.99	0.96	0.76
	Mortality	439	death	mortality	rate	births	animals	plants	years	areas	cause	populations
		WP	3.08	2.08	1.51	1.21	0.80	0.61	0.58	0.58	0.56	0.56
**MEDICAL**	Pharmaceutical	789	medicinal	plants	products	aromatic	crops	using	fruit	veterinary	vegetables	animal
		WP	3.80	2.81	2.43	1.37	1.21	1.03	0.92	0.92	0.84	0.79
	Healthcare	398	medical	services	areas	care	rural	healthcare	health	animals	development	region
		WP	4.10	1.74	1.09	1.06	1.03	1.00	0.74	0.68	0.68	0.61
**Macroc.**	**Category**	**Freq.** **Categ.**	**11**	**12**	**13**	**14**	**15**	**16**	**17**	**18**	**19**	**20**
**HEALTH**	Health	9037	protect.	develops	measur.	check	rural	quality	using	manag.	food	farms
		WP	0.57	0.57	0.54	0.54	0.53	0.51	0.46	0.44	0.42	0.42
	Unhealthy	12	problems	risk	supply	water	action	activities	agri	annoy	aspects	cereals
		WP	1.23	1.23	1.23	1.23	0.82	0.82	0.82	0.82	0.82	0.82
**SAFETY**	Safety	3489	agricult.	requirem.	protect.	conditions	standards	welfare	manag.	investm.	relative	equipment
		WP	0.83	0.78	0.77	0.73	0.71	0.67	0.55	0.54	0.52	0.52
	Toxic	479	agricult.	environ.	chemicals	soil	active	relating	also	organisms	levels	treatment
		WP	0.57	0.55	0.52	0.51	0.47	0.45	0.45	0.44	0.43	0.42
	Risk	36483	forests	applicat.	actions	nations	products	assessments	prevents	verifying	mitigation	system
		WP	0.53	0.52	0.50	0.49	0.44	0.39	0.37	0.37	0.37	0.32
	Prevention	3172	events	catastroph.	soils	caused	agricult.	restoration	supports	protective	improving	action
		WP	0.94	0.92	0.89	0.87	0.86	0.80	0.79	0.77	0.69	0.69
	Pathogens	287	spread	organis.	use	health	insects	risk	species	caused	crops	change
		WP	0.61	0.60	0.58	0.58	0.58	0.58	0.54	0.53	0.51	0.49
	Infection	132	fungal	mechanic	source	measures	reduce	plant	conditions	damage	parasitic	pests
		WP	0.62	0.58	0.55	0.51	0.51	0.47	0.44	0.44	0.44	0.44
	Hygiene	1423	health	investments	quality	food	requires	farms	protection	agricult.	livestock	working
		WP	1.09	1.04	0.98	0.91	0.83	0.77	0.71	0.57	0.55	0.55
	Epidemiology	73	animal	climate	prevention	water	development	increased	monitoring	studies	agricult.	farms
		WP	0.71	0.64	0.64	0.64	0.57	0.57	0.57	0.57	0.50	0.50
	Bacteria	133	soil	contamination	insects	management	area	bacterium	control	protective	quercus	defoliants
		WP	0.56	0.53	0.53	0.53	0.49	0.49	0.46	0.46	0.46	0.42
**DISEASES**	Disease	3509	including	control	nature	harmfulness	events	supported	description	environ.al	evidence	recommendation
		WP	0.84	0.82	0.81	0.80	0.78	0.74	0.73	0.70	0.70	0.69
	Single Diseases	71	improve	colorful	health	bark	processionaria	spread	castagno	ceratocystis	cinipide	cochineal
		WP	0.65	0.59	0.59	0.52	0.52	0.52	0.46	0.46	0.46	0.46
	Disability	1631	accession	programs	represents	equality	field	units	areas	accordance	discrimination	decision
		WP	0.73	0.70	0.69	0.69	0.67	0.63	0.58	0.54	0.53	0.52
	Mortality	439	natural	increase	protection	case	growth	agricult.	species	trees	rural	using
		WP	0.53	0.49	0.45	0.44	0.44	0.43	0.42	0.40	0.39	0.38
**MEDICAL**	Pharmaceutical	789	agricult.	food	drugs	herbs	areas	industry	protection	cultivation	farms	support
		WP	0.75	0.69	0.66	0.46	0.44	0.44	0.43	0.43	0.39	0.39
	Healthcare	398	social	infrastructure	public	community	supply	educational	school	populations	access	production
		WP	0.58	0.55	0.53	0.47	0.47	0.42	0.41	0.40	0.37	0.37

Note: WP: Weighted percentage.

**Table A6:** Word frequency within single Nutrition dimension categories.

**Macroc.**	**Category**	**Freq.** **Categ.**	**1**	**2**	**3**	**4**	**5**	**6**	**7**	**8**	**9**	**10**
**NUTRITION**	Obesity	6	obesity	territory	health	overweight	fact	plan	rdp	region	rural	
		WP	3.54	3.54	2.65	2.65	1.77	1.77	1.77	1.77	1.77	
	Malnutrition	4	freedom	free	malnutrition	animal	diseases	hunger				
		WP	5.81	4.65	4.65	3.49	3.49	2.33				
	Nutrition	590	nutritive	products	agricultural	quality	animal	food	health	using	crop	development
		WP	4.86	2.20	1.13	1.05	0.94	0.88	0.71	0.61	0.59	0.57
**DIETARY**	Individual_metabolic	343	intake	digestion	water	anaerobic	products	organic	use	plants	agricultural	nitrogen
		WP	3.50	1.61	1.03	0.84	0.83	0.77	0.75	0.73	0.68	0.65
	Eating	145	eats	products	animals	protection	plant	drinking	use	birds	areas	hands
		WP	4.05	1.56	1.03	0.93	0.86	0.83	0.80	0.76	0.73	0.70
	Diet	244	diets	products	cow	food	quality	health	dietary	costs	average	agricultural
		WP	4.31	2.41	1.23	1.12	0.75	0.43	0.67	0.65	0.60	0.56
**NUTRIENTS**	Micro_macronutr.	35	macronutrients	nitrogen	fertilizers	change	gross	soil	load	phosphorus	micronutrients	water
		WP	3.63	2.51	2.37	2.23	2.23	1.96	1.68	1.54	1.26	1.26
	Nutrient	3173	nutrients	waters	soil	reducing	balances	agricultural	areas	fertilizing	management	plants
		WP	5.38	2.00	1.75	1.02	0.89	0.86	0.82	0.77	0.73	0.70
	Iron	103	iron	island	rural	wood	activities	nitrate	water	wire	embroidery	lough
		WP	4.56	0.89	0.60	0.60	0.55	0.55	0.51	0.47	0.43	0.43
	Fat	731	fat	animal	product	pigs	breeds	cattle	farms	oils	milk	area
		WP	3.55	1.34	1.16	1.13	0.74	0.69	0.60	0.54	0.52	0.50
	Vitamin	9	vitamins	substances	minerals	food	high	antioxidants	feed	nutritional	acid	animal
		WP	5.39	3.59	2.99	2.40	2.40	1.80	1.80	1.80	1.20	1.20
	Sodium	15	sodium	areas	high	soil	development	nitrate	vertisol	agricultural	alkalizing	calcium
		WP	4.88	2.44	2.44	1.83	1.52	1.22	1.22	0.91	0.91	0.91
	Protein	538	proteins	crops	products	cereals	animals	oilseeds	farms	feed	plants	legumes
		WP	5.27	4.00	1.70	1.38	1.02	1.02	1.00	0.76	0.72	0.65
	Omega	7	omega	source	products	agriculture	emissions	milk	acids	cheese	dairy	equivalent
		WP	5.03	3.02	2.51	2.01	2.01	2.01	1.51	1.51	1.51	1.51
	Amino	4	protein	aminoacids	substitutes	birds	constant	ensure	fish	meal	plant	prunelli
		WP	5.16	3.23	3.23	1.94	1.94	1.94	1.94	1.94	1.94	1.94
	Calcium	60	calcium	nitrate	soil	potassium	magnesium	fertilizers	plant	organic	irrigation	carbonates
		WP	4.68	2.05	1.98	1.32	1.17	1.10	1.10	1.02	0.80	0.66
	Fiber	505	fibers	optical	infrastructures	network	areas	connectivity	broadband	region	plants	prescription
		WP	5.24	2.76	1.68	1.54	1.10	1.04	0.86	0.63	0.62	0.60
	Carbohydrate	10	carbohydrates	plant	reserves	increase	product	sugar	accelerate	altering	balance	center
		WP	5.78	2.89	2.31	1.73	1.73	1.73	1.16	1.16	1.16	1.16
**Macroc.**	**Category**	**Freq.** **Categ.**	**11**	**12**	**13**	**14**	**15**	**16**	**17**	**18**	**19**	**20**
**NUTRITION**	Obesity	6										
		WP										
	Malnutrition	4										
		WP										
	Nutrition	590	needs	promotion	areas	fertilizing	improving	soil	increase	values	measures	costs
		WP	0.55	0.53	0.53	0.48	0.47	0.47	0.46	0.46	0.43	48
**DIETARY**	Individual_metabolic	343	manure	energy	areas	including	soil	fertilizers	year	animal	reduced	management
		WP	0.63	0.57	0.56	0.53	0.51	0.48	0.48	0.47	0.43	0.43
	Eating	145	food	quality	requiring	species	development	new	activity	ensure	habits	livestock
		WP	0.66	0.66	0.66	0.63	0.56	0.56	0.53	0.50	0.50	0.47
	Diet	244	animals	breeds	farms’	market	yield	aid	increase	mediterranean	including	dairy
		WP	0.54	0.52	0.47	0.47	0.47	0.45	0.41	0.41	0.39	0.37
**NUTRIENTS**	Micro_macronutr.	35	indicator	use	balance	impact	needs	areas	natural	surface	unit	value
		WP	1.12	1.12	0.98	0.98	0.98	0.84	0.84	0.84	0.84	0.84
	Nutrient	3173	improvement	nitrogen	products	quality	crops	organisms	farms	lands	protects	measures
		WP	0.67	0.63	0.62	0.62	0.60	0.59	0.57	0.55	0.53	0.52
	Iron	103	area	development	products	traditional	craft	palma	systems	wine	interventions	level
		WP	0.38	0.38	0.38	0.38	0.34	0.34	0.34	0.34	0.30	0.30
	Fat	731	dairy	vegetal	meat	cows	poultry	agricultural	food	livestock	sectors	processing
		WP	0.46	0.44	0.43	0.41	0.31	0.29	0.27	0.27	0.27	0.26
	Vitamin	9	biological	content	conventional	favorable	harmful	organic	origin	products	quality	quantity
		WP	1.20	1.20	1.20	1.20	1.20	1.20	1.20	1.20	1.20	1.20
	Sodium	15	limit	measures	neutral	properties	acquisition	associated	based	biodiversity	change	chemical
		WP	0.91	0.91	0.91	0.91	0.61	0.61	0.61	0.61	0.61	0.61
	Protein	538	grain	use	agricultural	areas	food	sector	cultivation	vegetables	oil	wheat
		WP	0.58	0.58	0.56	0.53	0.53	0.50	0.48	0.45	0.44	0.44
	Omega	7	forestry	calcium	functional	health	higher	improvements	industries	lactose	oil	value
		WP	1.51	1.01	1.01	1.01	1.01	1.01	1.01	1.01	1.01	1.01
	Amino	4	tavignano	aminotriazole	ampa	animal	diet	glyphosate	optimum	quality	use	aims
		WP	1.94	1.29	1.29	1.29	1.29	1.29	1.29	1.29	1.29	0.65
	Calcium	60	fertilisers	phosphate	remove	gums	nutrient	preparations	acid	agrotextil	ammonium	mineral
		WP	0.66	0.66	0.59	0.51	0.51	0.51	0.44	0.44	0.44	0.44
	Fiber	505	operators	rural	services	access	technology	industrial	use	development	number	territory
		WP	0.55	0.54	0.54	0.51	0.50	0.49	0.46	0.45	0.45	0.43
	Carbohydrate	10	crops	decreasing	degradation	elements	evergreen	fungus	intake	internal	matter	methane
		WP	1.16	1.16	1.16	1.16	1.16	1.16	1.16	1.16	1.16	1.16

Note: WP: Weighted percentage.

**Table A7:** Word frequency within single Food dimension categories.

Macroc.	Category	Freq. Categ.	1	2	3	4	5	6	7	8	9	10
**FOOD**	Food	31855	food	agricultures	products	sectors	forestry	quality	developm.	industry	process.	promot.
		WP	5.05	3.95	2.96	1.32	1.20	1.08	0.99	0.98	0.80	0.74
	Attributes	4281	products	labels	traceability	quality	organized	systems	regulat.	agricultur.	using	seasonal
		WP	3.63	2.74	1.49	1.4	1.01	0.89	0.82	0.76	0.73	0.7
	Meal	253	meals	costs	accommodation	expenses	products	breakfast	expenditure	travel	replacement	bed
		WP	3.45	2.01	1.17	1.13	1	0.93	0.91	0.77	0.75	0.71
	Organic	826	products	organic	foods	agricultural	farms	regulations	quality	market	developm.	council
		WP	6.22	6.18	5.07	2.85	1.63	1.19	1.17	0.79	0.68	0.59
**CONSUMPTION**	Consumer behav.	496	consumers	products	purchase	awareness	quality	increase	agricultural	equipment	promotion	market
		WP	5.32	3.28	3.16	1.73	1.53	1.47	0.83	0.82	0.74	0.73
	Consumption	10929	consumption	products	energy	agriculture	waters	quality	increase	markets	foods	using
		WP	5.17	3.07	1.52	1.15	0.93	0.79	0.65	0.65	0.62	0.56
	Marketing	3889	agriculture	foods	products	promotions	markets	organizations	chain	processing	quality	managers
		WP	6.26	4.93	4.56	3.93	3.92	2.47	2.22	2.18	1.97	1.61
**FOOD** **SECUR.**	Food security	307	food	security	products	quality	improving	agriculture	environmental	health	systems	animal
		WP	6.19	5.14	2.29	1.52	1.15	1.14	0.9	0.85	0.69	0.67
**FOOD** **CHAIN**	Chain	9899	chains	products	food	agriculture	supply	markets	promotional	development	local	processing
		WP	5.34	3.67	1.99	1.9	1.9	1.73	1.46	1.07	1.03	1.02
	Market channel	1979	storing	retail	products	markets	foods	agricultural	services	carbon	using	sales
		WP	2.92	2.23	1.71	1.16	0.71	0.64	0.56	0.55	0.53	0.51
**Macroc.**	**Category**	**Freq.** **Categ.**	**11**	**12**	**13**	**14**	**15**	**16**	**17**	**18**	**19**	**20**
**FOOD**	Food	31855	markets	areas	rural	chains	improving	support	measuring	value	managing	schemes
		WP	0.62	0.61	0.61	0.57	0.56	0.52	0.51	0.47	0.46	0.45
	Attributes	4281	protection	market	controls	food	indicators	council	measuring	improvem.	process	develops
		WP	0.56	0.56	0.54	0.51	0.48	0.46	0.46	0.45	0.44	0.44
	Meal	253	farms	food	including	services	lunch	units	relating	used	accordance	dinner
		WP	0.66	0.66	0.64	0.62	0.53	0.53	0.49	0.47	0.46	0.46
	Organic	826	increase	areas	processing	measures	industry	schemes	sector	promotion	indicators	demand
		WP	0.52	0.51	0.51	0.49	0.46	0.44	0.40	0.39	0.39	0.37
**CONSUMPTION**	Consumer behav.	496	food	information	costs	activity	materials	use	environmental	energy	improve	change
		WP	0.70	0.64	0.64	0.60	0.55	0.54	0.52	0.50	0.48	0.46
	Consumption	10929	developments	areas	investments	regional	measuring	sectors	reducing	improve	promotional	systems
		WP	0.55	0.48	0.44	0.43	0.41	0.41	0.41	0.4	0.39	0.38
	Marketing	3889	including	animal	risk	sector	producers	welfare	local	schemes	value	support
		WP	1.58	1.47	1.39	1.37	1.36	1.33	1.13	1.07	1.02	0.75
**FOOD** **SECUR.**	Food security	307	safety	marketing	investments	disease	environment	promote	developm.	processing	traceability	well
		WP	0.67	0.66	0.62	0.59	0.56	0.56	0.54	0.53	0.53	0.48
**FOOD** **CHAIN**	Chain	9899	value	integrators	quality	improving	short	organized	management	projects	sectors	competitiv.
		WP	0.96	0.95	0.9	0.85	0.84	0.82	0.7	0.63	0.57	0.57
	Market channel	1979	areas	processing	trading	developments	systems	management	sector	rural	waters	protection
		WP	0.48	0.46	0.45	0.44	0.44	0.43	0.42	0.4	0.39	0.38

Note: WP: Weighted percentage.

## Appendix D

**Table A8:** Health dimension categories co-occurrences.

	HEALTH_DISEASE\Disability	HEALTH_DISEASE\Disease	HEALTH_DISEASE\Mortality	HEALTH_DISEASE\Single_Diseases	HEALTH_HEALTH\Health	HEALTH_HEALTH\Unhealth	HEALTH_MEDICAL\Medical_Healthcare	HEALTH_MEDICAL\Pharmaceutic	HEALTH_SAFETY\Bacteria	HEALTH_SAFETY\Epidemiological	HEALTH_SAFETY\Hygiene	HEALTH_SAFETY\Infection	HEALTH_SAFETY\Pathogens	HEALTH_SAFETY\Prevention	HEALTH_SAFETY\Risk	HEALTH_SAFETY\Safety	HEALTH_SAFETY\Tox
**HEALTH_DISEASE\Disability**	1631	16	12	0	68	0	9	3	0	0	0	0	0	15	16	4	0
**HEALTH_DISEASE\Disease**	1.0%	3509	40	32	409	0	5	31	27	22	44	65	48	893	619	50	7
**HEALTH_DISEASE\Mortality**	0.7%	1.1%	439	8	31	0	5	2	2	0	2	5	6	7	21	5	2
**HEALTH_DISEASE\Single_Diseases**	0.0%	0.9%	1.8%	71	10	0	0	3	3	0	2	4	1	16	5	0	0
**HEALTH_HEALTH\Health**	4.2%	11.7%	7.1%	14.1%	9037	4	156	55	7	24	331	21	50	292	804	684	55
**HEALTH_HEALTH\Unhealth**	0.0%	0.0%	0.0%	0.0%	0.0%	12	0	0	0	0	1	0	0	0	4	0	0
**HEALTH_MEDICAL\Medical_Healthcare**	0.6%	0.1%	1.1%	0.0%	1.7%	0.0%	398	14	0	2	8	1	0	6	10	6	0
**HEALTH_MEDICAL\Pharmaceutic**	0.2%	0.9%	0.5%	4.2%	0.6%	0.0%	3.5%	789	0	0	12	5	1	13	25	12	5
**HEALTH_SAFETY\Bacteria**	0.0%	0.8%	0.5%	4.2%	0.1%	0.0%	0.0%	0.0%	133	0	2	2	5	9	10	3	1
**HEALTH_SAFETY\Epidemiological**	0.0%	0.6%	0.0%	0.0%	0.3%	0.0%	0.5%	0.0%	0.0%	73	1	2	2	7	25	3	0
**HEALTH_SAFETY\Hygiene**	0.0%	1.3%	0.5%	2.8%	3.7%	8.3%	2.0%	1.5%	1.5%	1.4%	1423	4	3	29	36	366	2
**HEALTH_SAFETY\Infection**	0.0%	1.9%	1.1%	5.6%	0.2%	0.0%	0.3%	0.6%	1.5%	2.7%	0.3%	132	5	12	10	2	0
**HEALTH_SAFETY\Pathogens**	0.0%	1.4%	1.4%	1.4%	0.6%	0.0%	0.0%	0.1%	3.8%	2.7%	0.2%	3.8%	287	49	54	6	3
**HEALTH_SAFETY\Prevention**	0.9%	25.4%	1.6%	22.5%	3.2%	0.0%	1.5%	1.6%	6.8%	9.6%	2.0%	9.1%	17.1%	8958	3172	91	2
**HEALTH_SAFETY\Risk**	1.0%	17.6%	4.8%	7.0%	8.9%	33.3%	2.5%	3.2%	7.5%	34.2%	2.5%	7.6%	18.8%	35.4%	36483	154	44
**HEALTH_SAFETY\Safety**	0.2%	1.4%	1.1%	0.0%	7.6%	0.0%	1.5%	1.5%	2.3%	4.1%	25.7%	1.5%	2.1%	1.0%	0.4%	3489	5
**HEALTH_SAFETY\Tox**	0.0%	0.2%	0.5%	0.0%	0.6%	0.0%	0.0%	0.6%	0.8%	0.0%	0.1%	0.0%	1.0%	0.0%	0.1%	0.1%	479

Notes: In the right and high triangle, numbers are the co-occurrences between categories; in the left and low triangle, percentages represent the category co-occurrences on the total category occurrences (for example: total safety and health categories’ co-occurrence—684—on the total safety category occurrences—9037—equal to 7.6%).

**Table A9:** Nutrition dimension categories co-occurrences.

	DIET	EAT	INDIVIDUAL_METABOLIC	AMINO	CALCIUM	CARBOHYDRATE	FAT	FIBER	IRON	MICRO_MACRONUTRIENT	NUTRIENT	OMEGA	PROTEIN	SODIUM	VITAMIN	MALNUTRITION	NUTRITION	OBESITY
**DIET**	244	2	3	2	1	0	4	7	1	0	1	0	3	0	0	0	7	1
**EAT**	0.8%	145	0	0	0	0	0	0	0	0	2	0	0	0	0	0	7	0
**INDIVIDUAL_METABOLIC**	1.2%	0.0%	343	0	0	2	1	2	0	2	15	0	2	0	0	0	9	0
**AMINO**	0.8%	0.0%	0.0%	4	0	0	0	0	0	0	0	0	3	0	0	0	0	0
**CALCIUM**	0.4%	0.0%	0.0%	0.0%	60	0	0	0	2	1	11	2	0	4	0	0	1	0
**CARBOHYDRATE**	0.0%	0.0%	0.6%	0.0%	0.0%	10	1	0	0	0	0	0	1	0	0	0	0	0
**FAT**	1.6%	0.0%	0.3%	0.0%	0.0%	10.0%	731	4	0	0	6	1	24	0	1	0	10	0
**FIBER**	2.9%	0.0%	0.6%	0.0%	0.0%	0.0%	0.5%	505	2	0	2	0	6	0	1	0	1	0
**IRON**	0.4%	0.0%	0.0%	0.0%	3.3%	0.0%	0.0%	0.4%	103	0	1	0	0	1	0	0	0	0
**MICRO_MACRONUTRIENT**	0.0%	0.0%	0.6%	0.0%	1.7%	0.0%	0.0%	0.0%	0.0%	35	4	0	0	0	0	0	1	0
**NUTRIENT**	0.4%	1.4%	4.4%	0.0%	18.3%	0.0%	0.8%	0.4%	1.0%	11.4%	3173	0	5	0	0	0	57	0
**OMEGA**	0.0%	0.0%	0.0%	0.0%	3.3%	0.0%	0.1%	0.0%	0.0%	0.0%	0.0%	7	0	0	1	0	0	0
**PROTEIN**	1.2%	0.0%	0.6%	75.0%	0.0%	10.0%	3.3%	1.2%	0.0%	0.0%	0.2%	0.0%	538	0	0	0	10	0
**SODIUM**	0.0%	0.0%	0.0%	0.0%	6.7%	0.0%	0.0%	0.0%	1.0%	0.0%	0.0%	0.0%	0.0%	15	0	0	0	0
**VITAMIN**	0.0%	0.0%	0.0%	0.0%	0.0%	0.0%	0.1%	0.2%	0.0%	0.0%	0.0%	14.3%	0.0%	0.0%	9	0	3	0
**MALNUTRITION**	0.0%	0.0%	0.0%	0.0%	0.0%	0.0%	0.0%	0.0%	0.0%	0.0%	0.0%	0.0%	0.0%	0.0%	0.0%	4	0	0
**NUTRITION**	2.9%	4.8%	2.6%	0.0%	1.7%	0.0%	1.4%	0.2%	0.0%	2.9%	1.8%	0.0%	1.9%	0.0%	33.3%	0.0%	590	1
**OBESITY**	0.4%	0.0%	0.0%	0.0%	0.0%	0.0%	0.0%	0.0%	0.0%	0.0%	0.0%	0.0%	0.0%	0.0%	0.0%	0.0%	0.2%	6

Notes: In the right and high triangle, numbers are the co-occurrences between categories; in the left and low triangle, percentages represent the category co-occurrences on the total category occurrences (for example: total nutrition and diet categories’ co-occurrence—7—on the total diet category occurrences—244—equal to 2.9%).

**Table A10:** Food dimension categories co-occurrences.

	ATTRIBUTES	CHAIN	CHANNEL	CONSUMER	CONSUMER BEHAVIOUR	FOOD	FOOD MARKETING	FOOD SECURITY	MEALS	ORGANIC
**ATTRIBUTES**	4281	243	43	308	25	663	70	40	6	79
**CHAIN**	5.7%	9899	174	710	35	4374	1922	22	6	50
**CHANNEL**	1.0%	1.8%	1979	135	16	326	38	2	3	21
**CONSUMER**	7.2%	7.2%	6.8%	10,929	517	2105	170	40	17	112
**CONSUMER BEHAVIOR**	0.6%	0.4%	0.8%	4.7%	496	137	20	4	4	8
**FOOD**	15.5%	44.2%	16.5%	19.3%	27.6%	31,855	4161	314	40	864
**FOOD MARKETING**	1.6%	19.4%	1.9%	1.6%	4.0%	13.1%	3889	31	2	61
**FOOD SECURITY**	0.9%	0.2%	0.1%	0.4%	0.8%	1.0%	0.8%	307	0	2
**MEALS**	0.1%	0.1%	0.2%	0.2%	0.8%	0.1%	0.1%	0.0%	253	0
**ORGANIC**	1.8%	0.5%	1.1%	1.0%	1.6%	2.7%	1.6%	0.7%	0.0%	826

Note: In the right and high triangle, numbers are the co-occurrences between categories; in the left and low triangle, percentages represent the category co-occurrences on the total horizontal category occurrences (for example, total food and chain category co-occurrences—4374—on the total chain category occurrences—9899, equal to 44,2%).
